# Comparative efficacy of transcranial magnetic stimulation on different targets in Parkinson’s disease: A Bayesian network meta-analysis

**DOI:** 10.3389/fnagi.2022.1073310

**Published:** 2023-01-04

**Authors:** Ke Dong, Xiaoxia Zhu, Wenwu Xiao, Chu Gan, Yulu Luo, Manying Jiang, Hanjun Liu, Xi Chen

**Affiliations:** ^1^Department of Rehabilitation Medicine, The First Affiliated Hospital, Sun Yat-sen University, Guangzhou, China; ^2^Guangdong Provincial Key Laboratory of Brain Function and Disease, Zhongshan School of Medicine, Guangzhou, China

**Keywords:** transcranial magnetic stimulation, Parkinson’s disease, stimulation targets, network meta-analyses, motor recovery

## Abstract

**Background/Objective:**

The efficacy of transcranial magnetic stimulation (TMS) on Parkinson’s disease (PD) varies across the stimulation targets. This study aims to estimate the effect of different TMS targets on motor symptoms in PD.

**Methods:**

A Bayesian hierarchical model was built to assess the effects across different TMS targets, and the rank probabilities and the surface under the cumulative ranking curve (SUCRA) values were calculated to determine the ranks of each target. The primary outcome was the Unified Parkinson’s Disease Rating Scale part-III. Inconsistency between direct and indirect comparisons was assessed using the node-splitting method.

**Results:**

Thirty-six trials with 1,122 subjects were included for analysis. The pair-wise meta-analysis results showed that TMS could significantly improve motor symptoms in PD patients. Network meta-analysis results showed that the high-frequency stimulation over bilateral M1, bilateral DLPFC, and M1+DLPFC could significantly reduce the UPDRS-III scores compared with sham conditions. The high-frequency stimulation over both M1 and DLPFC had a more significant effect when compared with other parameters, and ranked first with the highest SCURA value. There was no significant inconsistency between direct and indirect comparisons.

**Conclusion:**

Considering all settings reported in our research, high-frequency stimulation over bilateral M1 or bilateral DLPFC has a moderate beneficial effect on the improvement of motor symptoms in PD (high confidence rating). High-frequency stimulation over M1+DLPFC has a prominent beneficial effect and appears to be the most effective TMS parameter setting for ameliorating motor symptoms of PD patients (high confidence rating).

## Introduction

Parkinson’s disease (PD) is a slowly progressive and neurodegenerative disease that affects more than 6 million people worldwide (Tolosa et al., 2021). Aging is the leading risk factor in PD, and populations above 50 years old have a higher prevalence, approximately 4% (de Rijk et al., 2000). The incidence and prevalence of PD are increasing as the population ages, (Tysnes and Storstein, 2017) as well as aggravates by air pollution (Lee et al., 2016). The main neuropathological correlates of PD are the loss of dopaminergic neurons in the basal ganglia circuit (Dauer and Przedborski, 2003; Mullin and Schapira, 2015) and the accumulation of Lewy bodies containing α-synuclein (Braak et al., 2003). Patients with PD manifest both motor symptoms and non-motor symptoms, which include resting tremor, rigidity, bradykinesia, postural instability, depression, sleep disorder, cognitive deficit, etc., (Postuma et al., 2015). These dysfunctions not only hamper the patient’s activities but increase the costs to families and burdens to the society.

The primary protocol for the initial treatment of PD patients is Levodopa (Ahlskog, 2010). Other drugs are also developed for alleviating symptoms of PD (Goetz et al., 2005). However, the drug complications and Levodopa-induced dyskinesia (LID) will appear along with the progress of PD (Ahlskog and Muenter, 2001; Rao et al., 2006). In order to delay the progression, improve the patient’s tolerance and obtain a better therapeutic effect, non-pharmacological interventions are now considered as a significant part for the treatment of PD (InformedHealth.org [Internet], 2015). In clinical settings, the most featured and widely applied non-pharmacological strategies for PD include rehabilitation training and neuromodulation techniques (Heumann et al., 2014; Abbruzzese et al., 2016).

Transcranial magnetic stimulation (TMS) is a non-invasive neuromodulation technique that could induce altered cortical excitability and synaptic plasticity in local brain regions and ameliorates symptoms in PD patients (Helmich et al., 2006). There are numerous studies that have verified the treatment effectiveness of TMS on PD with diverse parameters and rendered comparable results (Hamada et al., 2008; Cohen et al., 2018; Chung et al., 2020; Aftanas et al., 2021). Nevertheless, it is challenging to interpret the outcomes and improve the effectiveness of TMS in the absence of common parameter standards. Yokoe et al. found that only high-frequency repetitive-TMS (rTMS) over primary motor area (M1) and supplementary motor area (SMA) could improve the motor symptoms in PD, while the effects of TMS over dorsolateral prefrontal cortex (DLPFC) were similar to sham condition (Yokoe et al., 2018). In another study, the amelioration of symptoms still were detected in 3 months follow-up after low-frequency rTMS was applied to DLPFC (Zhuang et al., 2020). The diversity in stimulation parameters contributes to these discrepancies. A question then arises about whether there are specified optimal TMS settings (such as stimulation frequency and targets) for different PD symptoms. Several studies have compared TMS effects between different frequencies (Chung et al., 2020) and targets (Hanoglu et al., 2020), but comprehensive comparisons across frequencies and targets are still lacking. The network meta-analysis (NMA) can estimate the therapeutic effects of multiple interventions by incorporating the results of direct and indirect comparisons (Lumley, 2002), and present the probability rankings of interventions. In this study, our purpose is to explore a plausible TMS protocol including frequencies and targets for PD motor symptoms through an evidence-based network meta-analysis and give us an insight into personalized treatment for PD patients.

## Methods

### Literature search strategy

The Cochrane Handbook for Systematic Reviews of Interventions and the PRISMA extension statement (Hutton et al., 2015) were followed in this NMA study. We mainly searched for literature that focused on the application of TMS in PD patients. The online databases including Medline, Embase, PubMed Central, and Web of Science were searched for articles published date to December 2021. The search specified *Participants* and *Interventions*. The searching strategies were built as follows: (Parkinson Disease OR Idiopathic Parkinson’s Disease OR “other MeSH entry terms”) AND (Transcranial Magnetic Stimulation OR Magnetic Stimulation, Transcranial OR “other MeSH entry terms”) AND (max sensitivity filters for controlled trials). See Supplementary Appendix for actual searching terms. The PROSPERO registration number is: CRD42022329110.

### Inclusion and exclusion criteria

Two independent authors screened the retrieved results by reviewing the titles, abstracts, and full texts of literature. Any disagreements were resolved with consensus after group discussions. Studies that investigated the effectiveness of TMS for improving motor and non-motor symptoms of PD were eligible for our NMA. The inclusion criteria were: (1) *Study types*: randomized controlled trials (RCTs) or crossover RCTs. (2) *Participants*: patients diagnosed with PD. (3) *Interventions*: the rTMS protocols with different parameters. (4) *Comparison/Control*: the comparison among different frequencies or targets or the control were sham stimulation or active control. (5) *Outcomes*: the measurement of motor symptoms for PD.

The exclusion criteria were: (1) patients with secondary Parkinsonism or Parkinson’s plus syndrome. (2) the interventions included other types of neuromodulation techniques, such as deep brain stimulation (DBS) or transcranial direct current stimulation (tDCS). The other modalities of TMS, including theta-burst stimulation (TBS) and quadripulse stimulation (QPS), were also excluded. (3) conference abstracts, reviews, and other no-RCTs trials. (4) studies did not offer sufficient data for calculation. (5) studies with low quality.

### Quality assessment

We performed the quality assessment of studies with the recommended Cochrane Risk of Bias Tool mean values (Higgins and Green, 2011), which rated studies as three levels (high risk, low risk, unclear) in six domains: (1) Selective bias, (2) Performance bias, (3) Detection bias, (4) Attrition bias, (5) Reporting bias, (6) Other bias. The CINeMA (Confidence In Network-Analysis) online Web^1^ was applied to evaluate the quality of evidence considering six domains: Within-study bias, Reporting bias, Indirectness, Imprecision, Heterogeneity, and Incoherence. Any disagreements were resolved with consensus after group discussions.

### Data collection

The characteristics of included studies were extracted and summarized as follows: authors, publication year, study design, sample size, rTMS protocols (including stimulation intensity, frequency, target areas, and sessions), the group’s assignment, and the outcome measurements.

The primary outcome for analysis was the Unified Parkinson’s Disease Rating Scale part III (UPDRS-III), with higher scores indicating severe motor symptoms. For continuous data, we used the mean and standard deviation (m ± sd) of the changes in measurements after TMS administration for data synthesis. When studies provided these values (m ± sd) at baseline and post-intervention respectively, we used the following formula to calculate the changes:

$$M_{c\,h\,a\,n\,g\,e}=M_{p\,o\,s\,t}-M_{b\,a\,s\,e\,l\,i\,n\,e}$$

$$S\,D_{c\,h\,a\,n\,g\,e}=\sqrt{S\,D_{b\,a\,s\,e\,l\,i\,n\,e}^{2}+S\,D_{p\,o\,s\,t}^{2}-2\cdot C\,o\,r\,r\cdot S\,D_{b\,a\,s\,e\,l\,i\,n\,e}\cdot S\,D_{p\,o\,s\,t}}$$

The correlation coefficient (Corr) was calculated from the literature providing all the above indicators by the same formula. We used WebPlotDigitizer^2^ to extract numerical data when results were presented with figures. Standard errors and interquartile ranges were also used to obtain target data. We used the data at the end of all rTMS sessions for the results reported at multiple time points. The follow-up period without rTMS was not considered. The data extraction process was conducted under the guidance of the Cochrane handbook.

The stimulation frequency was extracted as high-frequency (hf) when it was greater than 1 Hz, or low-frequency (lf) when it was below or equal to 1 Hz. The unilateral target was extracted regardless of stimulating the left or right hemisphere. For some multiple stimulation targets studies, we described the targets as bilateral stimulation or one target area plus another area, including simultaneously or sequentially stimulated multiple targets. Any disagreements were resolved with consensus after group discussions.

### Network meta-analysis

We performed this network meta-analysis based on the Bayesian hierarchical models (Lu and Ades, 2004). The network graph was plotted using STATA software version 16.0 (Stata Corp, College Station, Texas, USA) to present the evidence of direct and indirect comparisons between interventions. We used the gemtc (*v.1.0-1*) (Valkenhoef and Kuiper, 2021) and rjags (*v.4-12*) (Plummer, 2022) software packages to establish a consistency model and conduct subsequent analysis. These two packages were based on the Markov Chain Monte Carlo method (MCMC) and included in R software *(v.4.1.2, R Foundation for Statistical Computing, Vienna, Austria)* (R Core Team, 2022). The model parameters were set as a random effect model with MCMC number of chains: 4, tuning iterations: 30,000, simulation iterations: 70,000, thinning interval: 1, variance scaling factor: 2.5. The model convergence was assessed with the Brooks-Gelman-Rubin diagnosis plot and the Potential Scale Reduction Factor (PSRF) (Brooks and Gelman, 1998; Tunaru, 2016).

The node-splitting approach was used to assess inconsistency by comparing estimates from both direct and indirect evidence (van Valkenhoef et al., 2016). The heterogeneity among studies was evaluated with the *I*^2^ statistics. The random-effect model was adopted with *I*^2^ > 50% in the global heterogeneity. The Sensitivity analysis was conducted to explore the sources of heterogeneity by excluding one literature at a time. The meta-regression analysis was performed to investigate the effect of different covariates on pooled effects. Publication bias was assessed using funnel plots and Egger’s test.

Statistical effects were set to be significant at *p < 0.05*. Pooled effect sizes were reported with mean difference (MD) and 95% credible intervals (CrIs) or confidence interval (CI). Both pair-wise meta-analysis and network meta-analysis were analyzed and presented. The rankogram was used to report the probability ranking of different stimulation targets. We applied the surface under the cumulative ranking curve (SUCRA) values to assess the likelihood that the interventions rank the best (Salanti et al., 2011). The SUCRA value is between 0 and 1, SUCRA equals 1 indicates that the intervention is absolutely effective, and SUCRA equals 0 indicates that the intervention is absolutely ineffective (Cope and Jansen, 2013). The interventions with a higher SUCRA value mean having better treatment efficacy.

## Results

### Searching results and characteristics of literature

**FIGURE 1:**
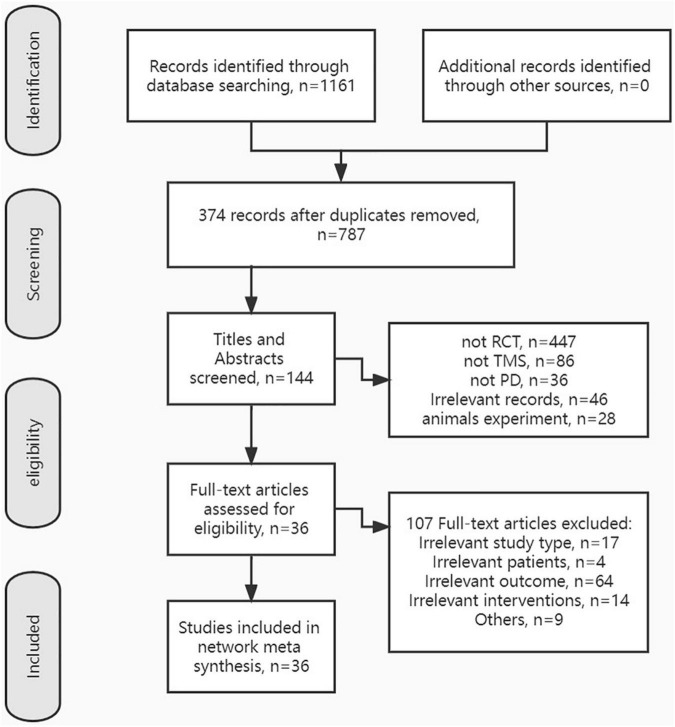
Flow chart presents the literature searching and screening process.

After removing duplicates, a total of 787 records were selected by titles and abstracts. Then, the full text of 144 articles was evaluated for eligibility. Finally, 36 RCTs on TMS improving motor symptoms of PD patients were included in this NMA. The exclusion reasons and the screening process are listed in Figure 1.

**FIGURE 2:**
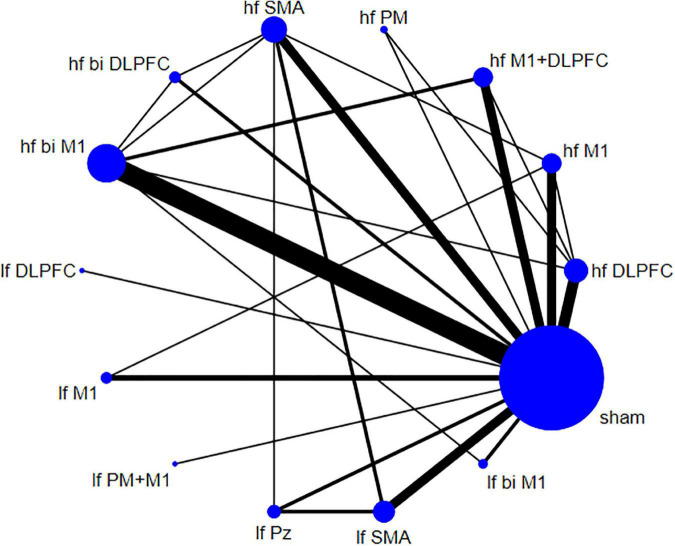
Network plot for Unified Parkinson’s Disease Rating Scale part III (UPDRS-III) shows the direct and indirect comparisons.

The descriptive data of 36 studies were presented in Table 1. A total of 36 English articles were included in our study, which contained 14 crossover trials (Börnke et al., 2004; Lefaucheur et al., 2004; Koch et al., 2005; Brusa et al., 2006; Rektorova et al., 2008; Filipović et al., 2009, 2010; Sedlácková et al., 2009; Maruo et al., 2013; Kim et al., 2015; Flamez et al., 2016; Dagan et al., 2017; Yokoe et al., 2018; Fricke et al., 2019) and 22 RCTs (Khedr et al., 2003; del Olmo et al., 2007; Hamada et al., 2008; Pal et al., 2010; Benninger et al., 2012; Shirota et al., 2013; Brys et al., 2016; Makkos et al., 2016; Shin et al., 2016; Aftanas et al., 2018; Cohen et al., 2018; Khedr et al., 2019; Mi et al., 2019; Randver et al., 2019; Chung et al., 2020; Hanoglu et al., 2020; Khedr et al., 2020; Li et al., 2020; Zhuang et al., 2020; Aftanas et al., 2021; Lench et al., 2021; Spagnolo et al., 2021). Of all publications, 24 articles (Khedr et al., 2003, 2019, 2020; Börnke et al., 2004; del Olmo et al., 2007; Hamada et al., 2008; Rektorova et al., 2008; Sedlácková et al., 2009; Pal et al., 2010; Benninger et al., 2012; Maruo et al., 2013; Kim et al., 2015; Brys et al., 2016; Makkos et al., 2016; Shin et al., 2016; Dagan et al., 2017; Aftanas et al., 2018, 2021; Yokoe et al., 2018; Mi et al., 2019; Randver et al., 2019; Hanoglu et al., 2020; Li et al., 2020; Spagnolo et al., 2021) used high-frequency stimulation (5–20 Hz), 7 articles (Brusa et al., 2006; Filipović et al., 2009, 2010; Flamez et al., 2016; Fricke et al., 2019; Zhuang et al., 2020; Lench et al., 2021) used low-frequency stimulation (0.1–1 Hz), 5 articles (Lefaucheur et al., 2004; Koch et al., 2005; Shirota et al., 2013; Cohen et al., 2018; Chung et al., 2020) compared the efficacy of both two frequencies. The stimulation targets involved unilateral or bilateral stimulation of M1, DLPFC, or both, and SMA, premotor cortex (PM), central parietal cortex (Pz), etc. All 36 studies set up comparisons with sham stimulation. The network plot depicted the direct and indirect comparisons among different rTMS targets (Figure 2). Node size and edge width were weighted by the involved sample size and number of studies.

**TABLE 1:** The characteristics of included studies.

References	Study design	Sample size		Parameters	Sessions	Assignment	Targets	Outcomes	Languages
		M	F						
Yokoe et al., 2018	crossover study	7	12	100% RMT; 10 Hz	3	rTMS(DLPFC)/rTMS(M1)/ rTMS(SMA)/sham	DLPFC-DLPFC//M1-M1//SMA(sequentially)	UPDRS-III	English
Spagnolo et al., 2021	parallel RCT	41	18	90–100%RMT; 10 Hz	12	rTMS(M1+PFC)/rTMS(M1)+ sham TMS(PFC)/sham(both)	bi M1-bi PFC//bi M1(sequentially)	UPDRS-III	English
Shin et al., 2016	parallel RCT	8	10	90% RMT; 5 Hz	10	real-rTMS/sham-rTMS	left DLPFC	UPDRS-III; HRS-D	English
Pal et al., 2010	parallel RCT	11	11	90% RMT; 5 Hz	10	real-rTMS/sham-rTMS	left DLPFC	UPDRS-II,III	English
Mi et al., 2019	parallel RCT	14	16	90% RMT; 10 Hz	10	real-rTMS/sham-rTMS	SMA	UPDRS-III	English
Maruo et al., 2013	crossover study	11	10	100% RMT; 10 Hz	3	real-rTMS/sham-rTMS	bi M1 foot area	UPDRS-III	English
Li et al., 2020	parallel RCT	16	32	80% RMT; 20 Hz	5	real-rTMS/sham-rTMS	M1 contralateral to pain site	UPDRS-III; HAMD	English
Lench et al., 2021	parallel RCT	14	6	110% RMT; 1 Hz	10	real-rTMS/sham-rTMS	SMA	UPDRS-III	English
Lefaucheur et al., 2004	crossover study	7	5	80% RMT; 0.5 Hz/10 Hz	1	0.5 Hz/10 Hz/sham/L-DOPA	left motor cortical area	UPDRS-III	English
Koch et al., 2005	crossover study	4	4	90% RMT; 1 Hz/110% RMT; 5 Hz	1	1 Hz/5 Hz/sham	SMA//Pz	UPDRS-III	English
Kim et al., 2015	crossover study	12	5	90% RMT; 10 Hz	5	real-rTMS/sham-rTMS	lower leg M1 of the dominant hemisphere	UPDRS-III	English
Khedr et al., 2019	parallel RCT	30		90% RMT; 20 Hz	10	real-rTMS/sham-rTMS	bi M1-hand area(sequentially)	UPDRS-III	English
Khedr et al., 2020	parallel RCT	24	9	90% RMT; 20 Hz	10	real-rTMS/sham-rTMS	bi M1-hand area(sequentially)	UPDRS-III; MoCA	English
Khedr et al., 2003	parallel RCT	24	12	120% MT; 5 Hz	10	real-rTMS/sham-rTMS	bi M1-hand area(sequentially)	UPDRS-III	English
Hamada et al., 2008	parallel RCT	54	44	110% AMT; 5 Hz	8	real-rTMS/sham-rTMS	SMA	UPDRS-III; HAMD	English
Filipović et al., 2010	crossover study	5	5	AMT; 1 Hz	4	real-rTMS/sham-rTMS	M1 contralateral to more severely side	UPDRS-III	English
del Olmo et al., 2007	parallel RCT	6	7	90% RMT; 10 Hz	10	real-rTMS/sham-rTMS	DLPFC contralateral to more affected side	UPDRS-III	English
Dagan et al., 2017	crossover study	7	0	100% RMT; 10 Hz	12/16	real-rTMS/sham-rTMS	H3 coil;bi medial PFC	UPDRS-III	English
Chung et al., 2020	parallel RCT	27	24	80% RMT; 1 Hz//80% RMT; 25 Hz	12	1 Hz/25 Hz/sham	bilateral M1 leg area(sequentially)	UPDRS-III	English
Brys et al., 2016	parallel RCT	37	24	RMT; 10 Hz	10	rTMS over bilateral M1 /left DLPFC/both/neither (sham rTMS)	bi M1-left DLPFC //bilateral M1//left DLPFC(sequentially)	UPDRS-III; HAMD	English
Brusa et al., 2006	crossover study	6	4	90% RMT; 1 Hz	1	L-DOPA/sham TMS/SMA rTMS/Pz rTMS	SMA//Pz	UPDRS-III	English
Benninger et al., 2012	parallel RCT	20	6	80% AMT;50 Hz	8	real-rTMS/sham-rTMS	bilateral M1	UPDRS-III	English
Aftanas et al., 2021	parallel RCT	21	25	100% RMT; 10 Hz//110% RMT; 10 Hz	20	real-rTMS/sham-rTMS	bilateral M1-LL-left DLPFC(sequentially)	UPDRS-II,III; HDRS-17	English
Zhuang et al., 2020	parallel RCT	18	15	110% RMT;1 Hz	10	real-rTMS/sham-rTMS	right DLPFC	UPDRS-III; HRS-D;PSQI; MoCA	English
Sedlácková et al., 2009	crossover study	9	1	100% RMT; 10 Hz	1	left PMd/left DLPFC /left OCC(control)	left PMd//left DLPFC //left OCC(control)	UPDRS-III	English
Rektorova et al., 2008	crossover study	5	1	90% RMT; 10 Hz	1	DLPFC/M1	left DLPFC//M1 contralateral to frequently used foot	UPDRS-III	English
Randver et al., 2019	parallel RCT	3	3	80% RMT; 10 Hz	6	3 W sham+3 W active/6 W active	left DLPFC	UPDRS-II,III; MoCA	English
Makkos et al., 2016	parallel RCT	24	20	90% RMT; 5 Hz	10	real-rTMS/sham-rTMS	bilateral M1	UPDRS-II,III; MoCA	English
Fricke et al., 2019	crossover study	15	5	95% RMT; 1 Hz	1	real ADS-rTMS/sham	(PMd+M1) contralateral to more affected body	UPDRS-III	English
Flamez et al., 2016	crossover study	8	7	90% RMT; 1 Hz	1/10	Single session(real/sham)/Multiple session(real/sham)	bi M1	UPDRS-III	English
Filipović et al., 2009	crossover study	5	5	AMT; 1 Hz	4	real-rTMS/sham-rTMS	M1 contralateral to the more severely affected side	UPDRS-III	English
Cohen et al., 2018	parallel RCT	32	10	110% MT; 1 Hz//100% MT; 10 Hz	24	real-rTMS/sham-rTMS	unilateral M1-bilateral DLPFC(sequentially)	UPDRS-III	English
Börnke et al., 2004	crossover study	6	6	90% RMT; 10 Hz	1	placebo+sham/placebo+ rTMS/Levodopa intake+rTMS/Levodopa intake+sham	M1 hand area contralateral to the severely affected side	UPDRS-III	English
Shirota et al., 2013	parallel RCT	45	61	110% RMT; 1 Hz//110% RMT; 10 Hz	8	1 Hz/10 Hz/sham	SMA	UPDRS-III; HAMD	English
Aftanas et al., 2018	parallel RCT	23	26	100% RMT; 10 Hz//110% RMT; 10 Hz	20	real-rTMS/sham-rTMS	bilateral M1-left DLPFC(sequentially)	UPDRS-III	English
Hanoglu et al., 2020	parallel RCT	11	5	100% MT; 5 Hz	10	left M1-rTMS/left SMA-rTMS	left M1//left SMA	UPDRS-III	English

ADS-rTMS, associative dual-site rTMS; AMT, active motor threshold; bi, bilateral; DLPFC, dorsolateral prefrontal cortex; HAMD/HDRS-17, Hamilton Rating Scale for Depression; HF, high frequency; i/c-TBS, intermittent/continuous-theta-burst stimulation; L-DOPA, levodopa; LF, low frequency; M1, primary motor area; MoCA, montreal cognitive assessment; NR, not reported; OCC, occipital cortex; PMd/PMC, dorsal premotor cortex/premotor cortex; PSQI, Pittsburgh sleep quality index; RCT, randomized controlled trial; RMT, resting motor threshold; rTMS, repetitive transcranial magnetic stimulation; SMA, supplementary motor area; UPDRS, Unified Parkinson’s Disease Rating Scale; Cz/Pz/C3, the electrode locations of standard 10–20 international system.

### Assessment of risk of bias and evidence grading

**FIGURE 3:**
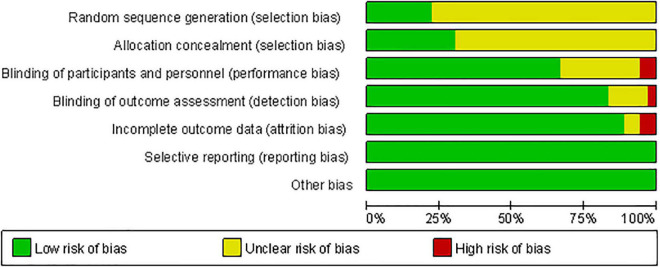
Risk of bias graph presents the quality of included studies.

Figure 3 shows the methodological quality assessment of the included studies. All studies used randomization, but 77.78% of studies did not report the method of generating random sequences, and 69.44% of studies had an unclear risk of allocation concealment. Two studies had participants lost to follow-up, making data incomplete (Brys et al., 2016; Cohen et al., 2018). Three studies only achieved single blindness in participants (Filipović et al., 2009, 2010) or investigators (Zhuang et al., 2020). All studies have no Reporting bias and Other bias. Overall, the quality of the included studies is moderate. The risk of bias in each study was summarized in Supplementary Figure 1. The CINeMA evidence grading results exhibited the confidence rating of all interventions relative to sham were available in Supplementary Figure 2. After accounting for all biases, the comparisons between *hf M1+DLPFC, hf bi DLPFC, hf bi M1*, and sham had high evidence grades. The comparisons with *hf PM, hf SMA, lf DLPFC, lf PM+M1, lf Pz* showed very low evidence grades. The comparisons with *hf DLPFC, hf M1, lf M1, lf Pz, lf SMA, lf bi M1* showed low to moderate evidence grades.

### Traditional pair-wise meta-analysis

**FIGURE 4:**
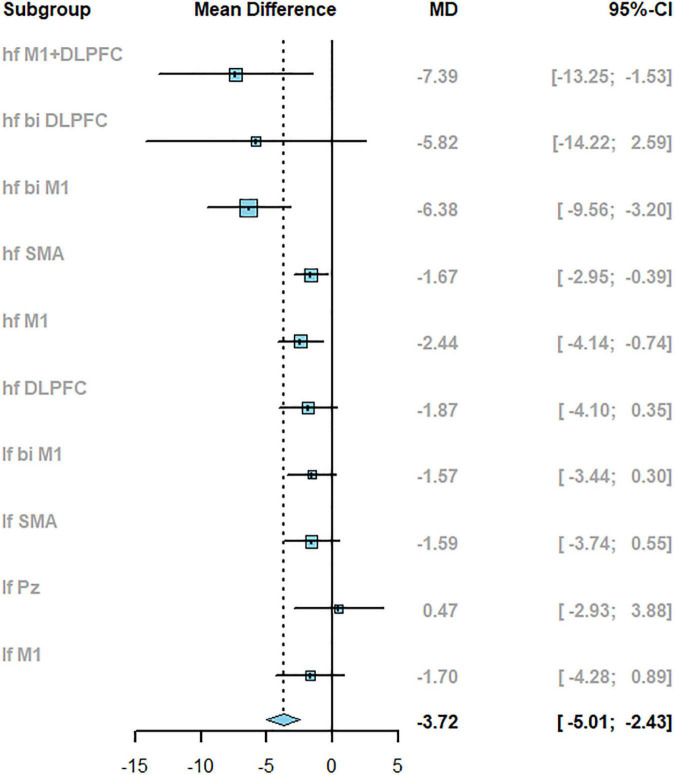
The results of pair-wise meta-analysis reported with mean difference (MD) and 95%CI when compared to sham.

The pair-wise meta-analysis investigated the efficacy of TMS with different parameters compared to control on PD (the inverse variance method with the random-effect model was used). As shown in Figure 4, TMS could significantly improve motor symptoms in PD patients compared with the control group (the total pooled effect sizes, MD: −3.72, 95% confidence intervals (CI): −5.01 to −2.43). Notably, the direct comparison results between active stimulation and sham condition indicated frequency- and target-dependent effects. The high-frequency stimulation targeting different areas showed a significant improvement in motor symptoms (MD: −4.63, 95% CI: −6.25 to −3.01), while low-frequency stimulation had not the same effects (MD: −1.21, 95% CI: −2.45 to 0.03). rTMS with *hf M1+DLPFC* induced the most significant improvement in UPDRS-III than other targets (MD: −7.39, 95% CI: −13.25 to −1.53).

### Network meta-analysis

Network meta-analysis estimates the efficacy of different rTMS targets in PD by incorporating evidence from direct and indirect comparisons. As exhibited in Table 2, the high-frequency stimulation over bilateral M1 (MD: −6.50, 95% CrI: −9.03 to −4.02), bilateral DLPFC (MD: −5.68, 95% CrI: −10.74 to −0.69), and M1+DLPFC (MD: −7.60, 95% CrI: −11.10 to −4.02) could significantly reduce the UPDRS-III scores when compared with sham condition. All low-frequency parameters were not effective in improving motor symptoms in PD patients. Concerning the comparison between active stimulations, the high-frequency stimulation over bilateral M1 had a better effect than targeting unilateral M1 (MD: −5.17, 95% CrI: −9.35 to −1.04). And, *hf M1+DLPFC* TMS showed more significant effects when compared to *hf M1* (MD: −6.27, 95% CrI: −11.07 to −1.40), *lf M1* (MD: −6.27, 95% CrI: −12.22 to −0.21), *lf SMA* (MD: −5.08, 95% CrI: −10.06 to −0.01), and *lf Pz* (MD: −6.46, 95% CrI: −12.67 to −0.17).

**TABLE 2:** The results of the network meta-analysis reported with mean difference (MD) and 95% CrI.

**hf bi M1**													
**−5.17 (−9.35, −1.04)**	**hf M1**												
−0.82 (−6.17,4.55)	4.35 (−1.58,10.37)	**hf bi DLPFC**											
−3.56 (−7.98,0.82)	1.61 (−3.19,6.43)	−2.74 (−9.06,3.50)	**hf DLPFC**										
−3.40 (−7.36,0.53)	1.77 (−2.52,6.11)	−2.57 (−8.18,3.01)	0.17 (−4.76,5.11)	**hf SMA**									
1.11 (−3.01,5.07)	**6.27 (1.40,11.07)**	1.93 (−4.28,7.98)	4.66 (−0.34,9.61)	4.50 (−0.33,9.22)	**hf M1+DLPFC**								
−4.05 (−12.13,4.02)	1.12 (−7.20,9.45)	−3.22 (−12.45,5.96)	−0.47 (−8.25,7.24)	−0.65 (−9.01,7.70)	−5.15 (−13.52,3.31)	**hf PM**							
−4.28 (-9.96,1.37)	0.89 (−5.47,7.26)	−3.45 (−10.81,3.84)	−0.71 (−7.30,5.87)	−0.88 (−7.15,5.38)	−5.38 (−11.73,1.10)	−0.24 (−9.64,9.18)	**lf bi M1**						
−5.16 (−10.66,0.28)	0.01 (−5.47,5.47)	−4.33 (−11.36,2.59)	−1.59 (−7.70,4.51)	−1.76 (−7.55,4.01)	**−6.27 (−12.22,-0.21)**	−1.11 (−10.20,7.95)	−0.87 (−8.13,6.35)	**lf M1**					
−0.99 (−10.05,8.04)	4.17 (−5.12,13.53)	−0.17 (−10.25,9.87)	2.55 (−6.89,12.10)	2.40 (−6.87,11.69)	−2.10 (−11.45,7.35)	3.05 (−8.57,14.62)	3.29 (−6.93,13.53)	4.16 (−5.74,14.14)	**lf DLPFC**				
−3.97 (−8.35,0.34)	1.19 (−3.63,6.01)	−3.16 (−9.25,2.89)	−0.41 (−5.63,4.79)	−0.59 (−4.83,3.67)	**−5.08 (−10.06, −0.01)**	0.07 (−8.46,8.55)	0.30 (−6.18,6.76)	1.17 (−4.84,7.22)	−2.98 (−12.41,6.40)	**lf SMA**			
−5.36 (−11.07,0.32)	−0.20 (−6.26,5.92)	−4.55 (−11.66,2.58)	−1.80 (−8.16,4.59)	−1.97 (−7.60,3.67)	**−6.46 (−12.67,-0.17)**	−1.32 (−10.61,7.97)	−1.08 (−8.56,6.40)	−0.21 (−7.25,6.88)	−4.36 (−14.44,5.77)	−1.39 (−6.65,3.92)	**lf Pz**		
−7.83 (−16.56,0.80)	−2.67 (−11.65,6.28)	−7.01 (−16.75,2.66)	−4.27 (−13.45,4.81)	−4.44 (−13.37,4.48)	−8.93 (−17.97,0.12)	−3.79 (−15.13,7.52)	−3.56 (−13.51,6.35)	−2.70 (−12.33,6.98)	−6.85 (−18.97,5.23)	−3.86 (−12.92,5.19)	−2.48 (−12.28,7.29)	**lf PM+M1**	
**−6.50 (−9.03,−4.02)**	−1.33 (−4.68,2.02)	**−5.68 (−10.74,−0.69)**	−2.93 (−6.73,0.84)	−3.10 (−6.36,0.14)	**−7.60 (−11.10,−4.02)**	−2.45 (−10.16,5.23)	−2.22 (−7.65,3.16)	−1.34 (−6.18,3.51)	−5.49 (−14.22,3.16)	−2.52 (−6.11,1.04)	−1.13 (−6.33,4.00)	1.34 (−6.95,9.66)	**sham**

Values in bold indicate statistically significant. For lower left triangle, the value comes from the NMA results of former parameters relative to the latter.

### Rank probability

**FIGURE 5:**
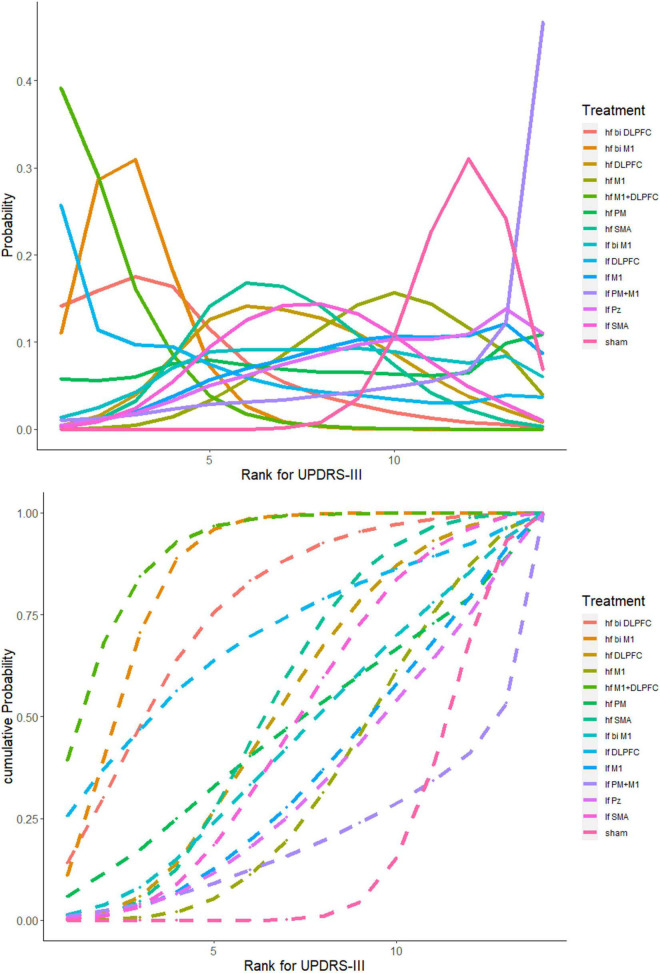
The rankogram and cumulative ranking plot. [**(Top)**: The rank probabilities of each parameter. **(Bottom)**: The cumulative ranking plot to present SUCRA value. The *hf M1+DLPFC* (green line) has the highest probability of being ranked first (39.14%) and had the highest SUCRA value (90.66%)].

For UPDRS-III, the probability of *hf M1+DLPFC* being best-ranked was 39.14% and had the highest SUCRA value (90.66%). The *hf bi M1* ranked third with a probability of 30.93%, and a SUCRA value was 84.90%. The *lf PM+M1* had the worst ranking probability of 46.70%, and a SCURA value was 19.38%. Figure 5 demonstrates the rankogram and cumulative ranking plot of different TMS targets.

### Model convergence, consistency, and bias of publication

Model convergence was assessed using the Brooks-Gelman-Rubin method by comparing within-chain and between-chain variance. The Brooks-Gelman-Rubin diagnostic plot revealed that the median value and 97.5% value of the reduction factor tend to be one after 70,000 iterations, and the PSRF value was also close to 1, which suggested a satisfactory convergence had been reached (Supplementary Figure 3). The node-splitting method revealed that there was no significant inconsistency between the direct and indirect comparisons (*p > 0.05*), which means the results of the used consistency model were reliable (Supplementary Figure 4). The heterogeneity analysis showed global heterogeneity with *I*^2^ > 50%. Therefore, the random effect model was used in both pair-wise meta-analysis and NMA. Sensitivity analysis was also performed by omitting one study at a time. The results of sensitivity analysis showed the robustness of pooled effects in the outcome. A meta-regression analysis using the sessions as a covariate indicated the rTMS session was negatively related to the improvement in UPDRS-III (i.e., the more sessions, the larger improvement in motor symptoms. See Supplementary Figure 5). However, the effect was not significant (the mean shared regression coefficient was −3.33, 95% CI: −6.87 to 0.30). The bias of publication was not observed in included studies, with a symmetrical funnel plot and *p* = 0.6727 in Egger’s test (Supplementary Figure 6).

## Discussion

A growing body of literature has investigated the therapeutic effects of TMS with different parameters on PD patients, but the majority of studies were compared with either sham or conventional interventions or another target. There was still a lack of literature to comprehensively estimate the efficacy of TMS on the treatment of PD with multiple different frequencies and targets. Accordingly, we conducted this NMA, which included 1,122 PD patients in 36 original studies. According to the TMS frequency and target involved in the articles, 14 TMS parameter settings were extracted and determined. The primary outcome was changes in UPDRS-III scores that evaluate the motor function of PD patients. The results of the pair-wise meta-analysis showed that different TMS parameters were more effective than control on improvement of motor symptoms of PD patients. After mixing with indirect evidence, high-frequency TMS targeting bilateral M1, bilateral DLPFC, and both M1 and DLPFC could significantly improve motor symptoms of PD patients. *Hf M1+DLPFC* was the most effective intervention among them.

The M1 is a pivotal brain area for generating voluntary movements and interacts with the basal ganglia through direct and indirect pathways (Galvan et al., 2015; Xu et al., 2017). The synchrony and excitability in the M1 could be affected by midbrain dopaminergic innervation (Parr-Brownlie and Hyland, 2005; Grandi et al., 2018). Different models including the Rate model, (Albin et al., 1989) Oscillation model, (Hammond et al., 2007), and plasticity model, (Wichmann, 2019) as well as neuroimaging results, (Burciu and Vaillancourt, 2018) give explanations for functional changes in M1 in PD patients. Therefore, motor dysfunction in PD patients often involves modifications in the physiological properties of neurons in M1 (Underwood and Parr-Brownlie, 2021). Studies have also shown that TMS could induce activity changes in M1 and normalize related neural network circuits, (Lomarev et al., 2006; González-García et al., 2011) and even lead to the release of dopamine in the striatum (Strafella et al., 2005).

The prefrontal cortex plays a crucial role in the distributed network of cognitive processing (Medaglia et al., 2015). In particular, the DLPFC is usually involved in inhibitory control, performance monitoring, action selection, and reward learning, (Ridderinkhof et al., 2004) can also modulate dopamine release in the striatum (Murase et al., 1993; Strafella et al., 2001). The intra-cortical connection between DLPFC and M1 can transfer crucial inhibitory stimulus to perform motor output (Cisek, 2006; Cohen et al., 2010). Impaired connectivity between the prefrontal cortex, PMC, and SMA is related to bradykinesia (Rowe et al., 2010; Wu et al., 2011). Several studies have demonstrated that the activity of DLPFC was decreased in PD patients (Schmiedt-Fehr et al., 2007; Singh et al., 2018; Trujillo et al., 2019).

Based on the above discussion, there are several possible explanations for why M1+DLPFC showed a better performance. First, facilitation of both regions may additionally increase the dopamine release of the striatal. Second, the better performance may originate from the superposition of simple stimulation in the two regions (dose-effect). Third, the M1 and DLPFC are hubs for neural communication in PD-related neural networks and are associated with symptoms such as bradykinesia, resting tremors, and cognition impairment (Gao and Wu, 2016). Cao et al. have found that the excitatory changes of DLPFC to M1 are bidirectional (Cao et al., 2018), and the connectivity of the cortical-basal ganglia-thalamo-cortical pathway is related to the alleviation of PD symptoms (DeLong and Wichmann, 2007; Tachibana et al., 2011). Thus, double stimulation may alter the interactions between different regions by activating cortical-cortical or cortical-subcortical networks, thereby facilitating information communication and action output. Forth, the dyskinesia of PD is related to the neurophysiological alterations in M1. Besides the allocation of cognitive resources in DLPFC, the compensatory responses from the motor to the cognitive system are also essential for optimal motor output (Cao et al., 2018; Dagan et al., 2018). The double stimulation may balance the allocation between the two regions. We also found that TMS over bilateral M1 was more effective than unilateral stimulation. Besides the bilateral communication mentioned above, it may be related to the possible bilateral dysfunction in PD.

Several pair-wise meta-analyses have evaluated the effect of TMS on motor and non-motor symptoms of PD (Chou et al., 2015; Zanjani et al., 2015; Zhu et al., 2015; Chung and Mak, 2016; Yang et al., 2018). Yet, to our knowledge, this NMA is the first time to compare the efficacy of different TMS parameters in PD. Unlike those direct comparisons, after mixing indirect evidence, we found that *hf M1+DLPFC* reduced UPDRS-III by −7.60 points (95% CrI: −11.10 to −4.02) compared to the sham condition and had a 90.66% probability of being better than other parameters. In addition, the effect size in our NMA is significantly larger than the degree of improvement of UPDRS-III in other meta-analyses [Elahi et al. with −6.68 points (95% CI: −9.66 to −3.69) (Elahi et al., 2009), Zhu et al. with −5.05 points (95% CI: −8.37 to −1.73) (Zhu et al., 2015)]. But it is close to the result of *hf bi M1* (MD: −6.56, 95% CrI: −9.10 to −4.09) and *hf bi DLPFC* (MD: −5.69, 95% CrI: −10.77 to −0.68) in our NMA. A previous study found −3.25 points for the Minimal Clinically Important Difference (MCID) in UPDRS-III (Horváth et al., 2015). This NMA demonstrated that *hf bi M1* and *hf bi DLPFC* have a moderate beneficial effect on UPDRS-III, and the effect of *hf M1+DLPFC* is more prominent. The possible explanations are described above. The finding that a combination of high-frequency stimulation and the target produced better effects may be attributed to the activity of the brain state and connectivity between regions (Chou et al., 2015). Researchers have found that high-frequency rTMS over the M1 and low-frequency rTMS over the DLPFC have stronger and more pronounced therapeutic effects. Similarly, low-frequency rTMS over SMA produced long-lasting beneficial effects, (Shirota et al., 2013). Still, the outcome was not significant when low-frequency rTMS was applied to bilateral M1 (Chung et al., 2020). This suggests that the activity of the target area may determine the effect of the frequency of stimulation used.

Our NMA yielded some interesting results, but several limitations need to be acknowledged. The cognition, mood, sleep disorders, and other non-motor symptoms were not considered. Another issue was that the medication status was not included, which probably had biased our results. The long-term effects of TMS were not evaluated since we only compared the difference between the baseline and immediately after the end of all TMS sessions. Studies targeting other regions, such as the cerebellum, brainstem, and spinal cord, were excluded because they did not fully meet the criteria and were not discussed. In addition, we did not distinguish stimulation order in multiple targets, and we defined both simultaneous and sequential stimulation patterns as bilateral or one target plus another, which might bias the interpretation. Finally, the characteristics of patients such as age, PD stage, and accompanying symptoms were also likely to confound our results and should be noted.

## Conclusion

The main conclusion drawn from our network meta-analysis is that TMS could significantly improve motor symptoms in PD patients. When considering all parameter settings, high-frequency stimulation targeting bilateral M1 or bilateral DLPFC has a moderate beneficial effect on improving motor symptoms in PD (high confidence rating). High-frequency stimulation over M1+DLPFC has a large beneficial effect and appears to be the most effective TMS parameter setting for ameliorating motor symptoms in PD patients (high confidence rating).

With the advancement of neuromodulation techniques, the individualized and precise TMS regulation on PD patients is essential to improving the therapeutic effect. Based on the evidence, this research proposes the optimal TMS frequency and targets for motor symptoms of PD patients. However, considering some limitations in this study, the large-scale and multi-center clinical trials are still irreplaceable in the future to demonstrate the relationship between different parameters and clinical outcomes.

## Data availability statement

The original contributions presented in this study are included in the article/Supplementary material, further inquiries can be directed to the corresponding authors.

## Author contributions

KD: conceptualization, methodology, software, writing—original draft, formal analysis, and visualization. XZ: conceptualization, methodology, writing—review and editing, formal analysis, and validation. WX, CG, YL, and MJ: writing—review and editing and validation. XC and HL: conceptualization, supervision, project administration, and funding acquisition. All authors contributed to the article and approved the submitted version.

## Footnotes

1
https://cinema.ispm.unibe.ch/

2
https://apps.automeris.io/wpd/index.zh_CN.html
