# Supplementary material for: Comparative efficacy of transcranial magnetic stimulation on different targets in Parkinson’s disease: A Bayesian network meta-analysis
Source: Front Aging Neurosci. 2023 Jan 4;14:1073310. doi: 10.3389/fnagi.2022.1073310 (PMC9845788; doi:10.3389/fnagi.2022.1073310)

**Supplementary Material**

**Contents**

[Appendix. The actual searching terms. 2](#_Toc121913053)

[Fig.S1 The Risk of bias summary shows the quality of each study. 4](#_Toc121913054)

[Fig.S2 The CINeMA evidence grading results of all parameters when compared with sham. 5](#_Toc121913055)

[Fig.S3 The Brooks-Gelman-Rubin diagnostic plot shows the model convergence. 6](#_Toc121913056)

[Fig.S4 The node-splitting method for inconsistency analysis by comparing the direct with indirect evidence. (All p > 0.05 indicate the inconsistency is not significant). 7](#_Toc121913057)

[Fig.S5 The meta-regression results shows the effects of stimulation sessions on pooled effect size. 9](#_Toc121913058)

[Fig.S6 The funnel plots to assess the bias of publication. 11](#_Toc121913059)

# Appendix. The actual searching terms.

**English** **databases.**

**PubMed**

..........................................................................................................................................

1 Parkinson Disease OR Idiopathic Parkinson's Disease OR Lewy Body Parkinson's Disease OR Parkinson's Disease, Idiopathic OR Parkinson's Disease, Lewy Body OR Parkinson Disease, Idiopathic OR Parkinson's Disease OR Idiopathic Parkinson Disease OR Lewy Body Parkinson Disease OR Primary Parkinsonism OR Parkinsonism, Primary OR Paralysis Agitans 129,518

2 Transcranial Magnetic Stimulation OR Magnetic Stimulation, Transcranial OR Magnetic Stimulations, Transcranial OR Stimulation, Transcranial Magnetic OR Stimulations, Transcranial Magnetic OR Transcranial Magnetic Stimulations OR Transcranial Magnetic Stimulation, Single Pulse OR Transcranial Magnetic Stimulation, Paired Pulse OR Transcranial Magnetic Stimulation, Repetitive 19,945

3 (randomized controlled trial [pt] OR controlled clinical trial [pt] OR randomized [tiab] OR placebo [tiab] OR drug therapy [sh] OR randomly [tiab] OR trial [tiab] OR groups [tiab]) NOT (animals [mh] NOT humans [mh]) 4,569,908

4 #1 AND #2 AND #3 289

**Cochrane library**

..........................................................................................................................................

Search Name: PD

Date Run: 14/11/2021 21:35:37

Comment: for META

ID Search Hits

#1 MeSH descriptor: [Parkinson Disease] explode all trees 4476

#2 MeSH descriptor: [Transcranial Magnetic Stimulation] explode all trees 1496

#3 #2 AND #1 67

**Web of Science**

..........................................................................................................................................

#1 246067 TS=(Parkinson Disease OR Idiopathic Parkinson’s Disease OR Lewy Body Parkinson’s Disease OR Parkinson’s Disease, Idiopathic OR Parkinson’s Disease, Lewy Body OR Parkinson Disease, Idiopathic OR Parkinson’s Disease OR Idiopathic Parkinson Disease OR Lewy Body Parkinson Disease OR Primary Parkinsonism OR Parkinsonism, Primary OR Paralysis Agitans)

#2 33886 TS=(Transcranial Magnetic Stimulation OR Magnetic Stimulation, Transcranial OR Magnetic Stimulations, Transcranial OR Stimulation, Transcranial Magnetic OR Stimulations, Transcranial Magnetic OR Transcranial Magnetic Stimulations OR Transcranial Magnetic Stimulation, Single Pulse OR Transcranial Magnetic Stimulation, Paired Pulse OR Transcranial Magnetic Stimulation, Repetitive)

#3 13151081 TS=clinical trial* OR TS=research design OR TS=comparative stud* OR TS=evaluation stud* OR TS=controlled trial* OR TS=follow-up stud* OR TS=prospective stud* OR TS=random* OR TS=placebo* OR TS=(single blind*) OR TS=(double blind*)

#4 572 #3 AND #2 AND #1

**Embase**

..........................................................................................................................................

No. Query Results Results Date

#1. ('parkinson disease'/exp OR 'lewy bodies of 233 14 Nov 2021

parkinson disease' OR 'lewy bodies of parkinson`s

disease' OR 'lewy bodies of parkinsons disease'

OR 'lewy body parkinson disease' OR 'lewy body

parkinson`s disease' OR 'lewy body parkinsons

disease' OR 'parkinson dementia complex' OR

'parkinson disease' OR 'parkinson`s disease' OR

'parkinsons disease' OR 'idiopathic parkinsonism'

OR 'paralysis agitans' OR 'primary parkinsonism')

AND ('transcranial magnetic stimulation'/exp OR

'magnetic stimulation, transcranial' OR

'stimulation, transcranial magnetic' OR

'transcranial magnetic stimulation') AND

('randomized controlled trial'/exp OR 'controlled

trial, randomized' OR 'randomised controlled

study' OR 'randomised controlled trial' OR

'randomized controlled study' OR 'randomized

controlled trial' OR 'trial, randomized

controlled')

# Fig.S1 The Risk of bias summary shows the quality of each study.

# Fig.S2 The CINeMA evidence grading results of all parameters when compared with sham.


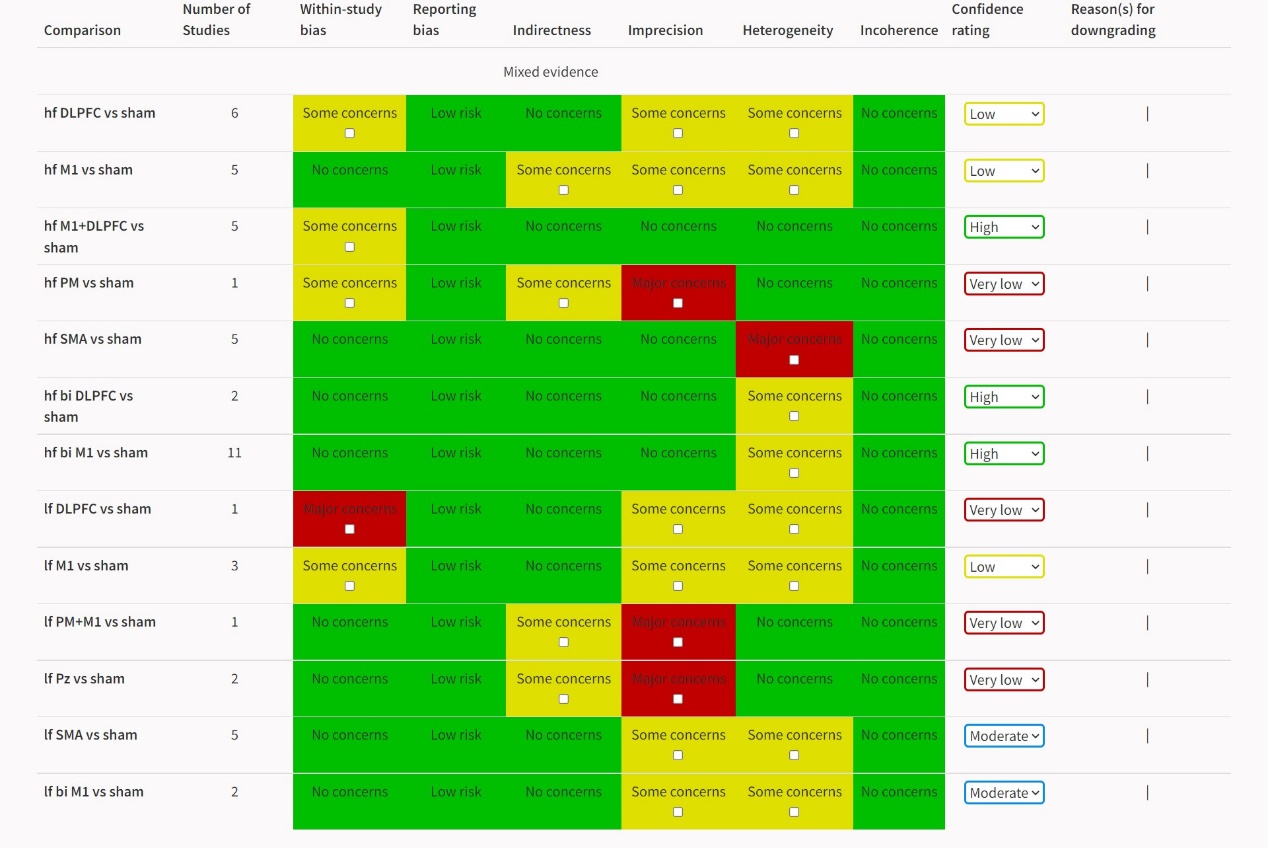


# Fig.S3 The Brooks-Gelman-Rubin diagnostic plot shows the model convergence.


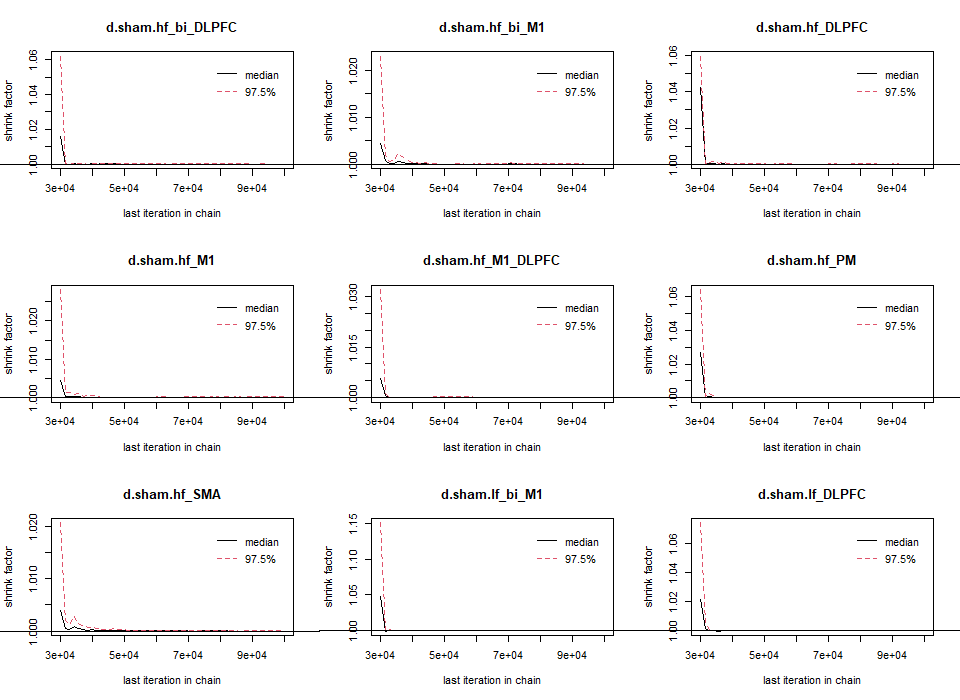


**
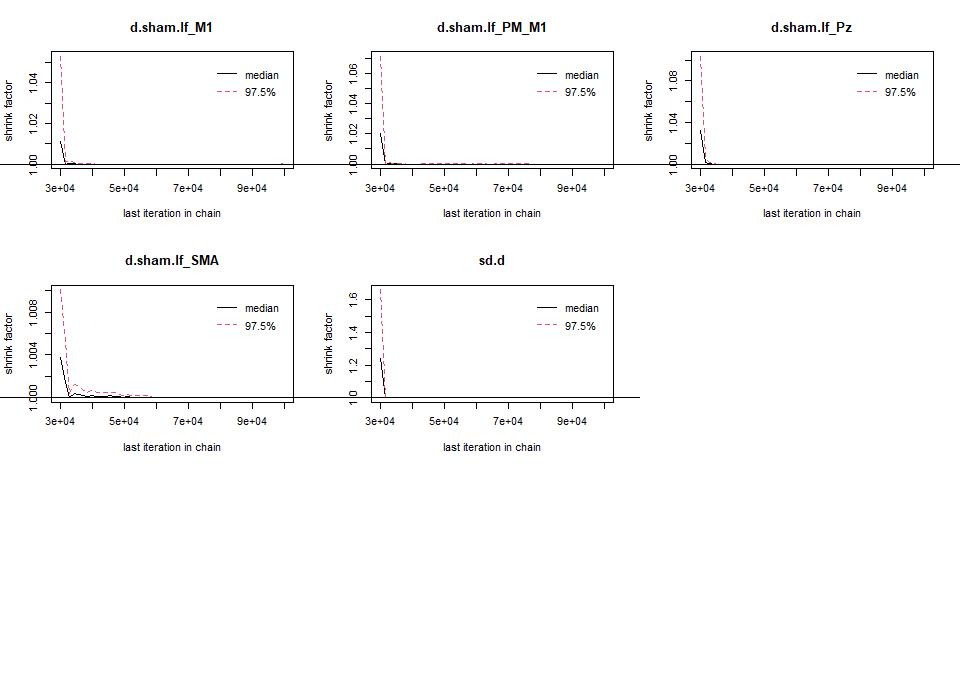
**

# Fig.S4 The node-splitting method for inconsistency analysis by comparing the direct with indirect evidence. (All p > 0.05 indicate the inconsistency is not significant).


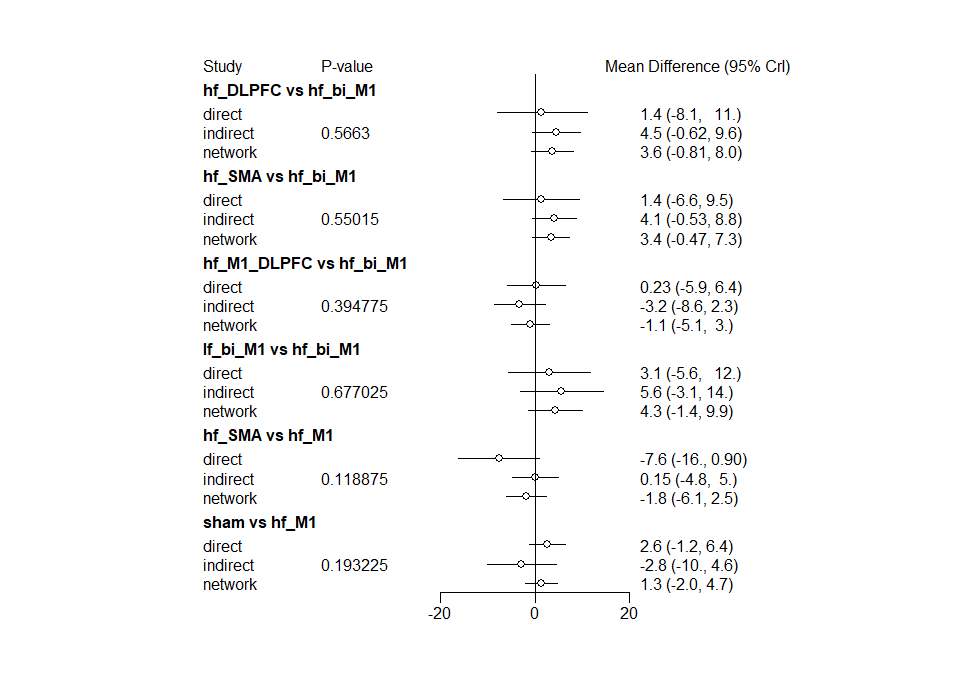


**
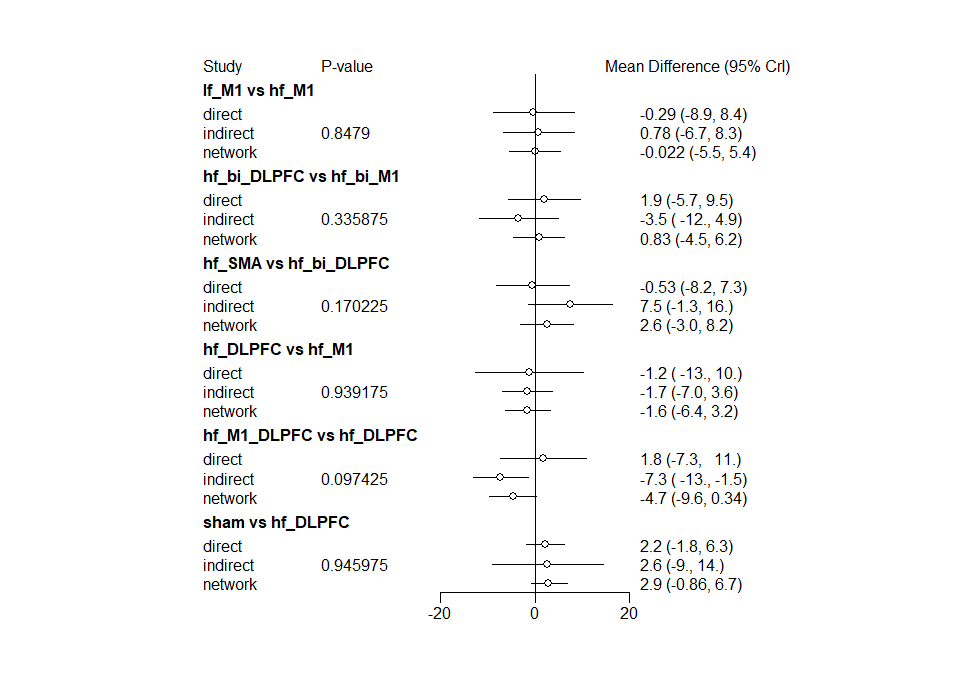
**

**
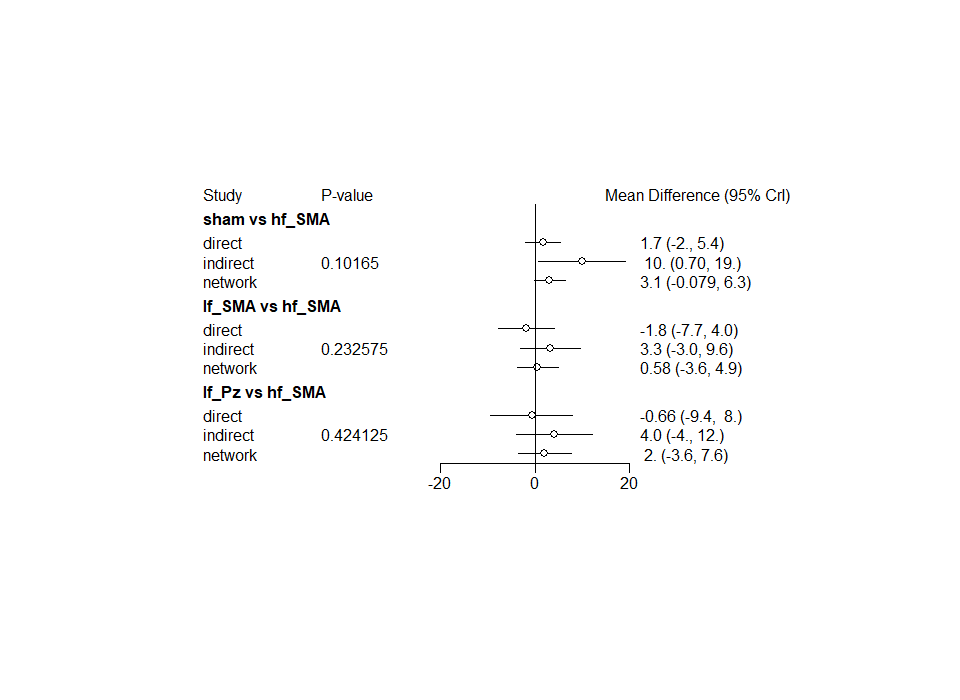
**

# Fig.S5 The meta-regression results shows the effects of stimulation sessions on pooled effect size.

**
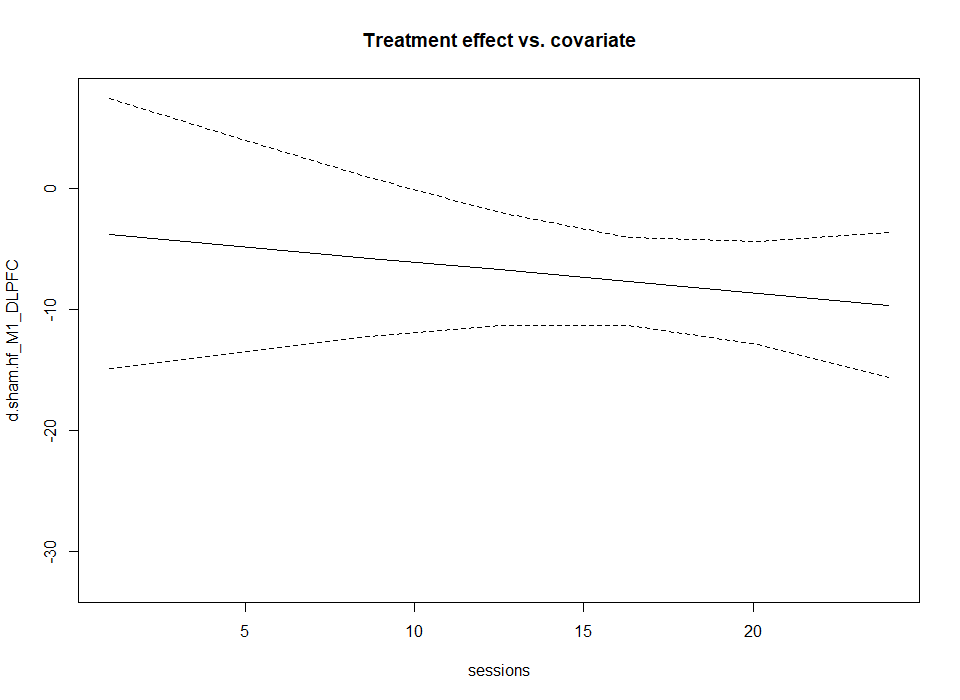
**

**
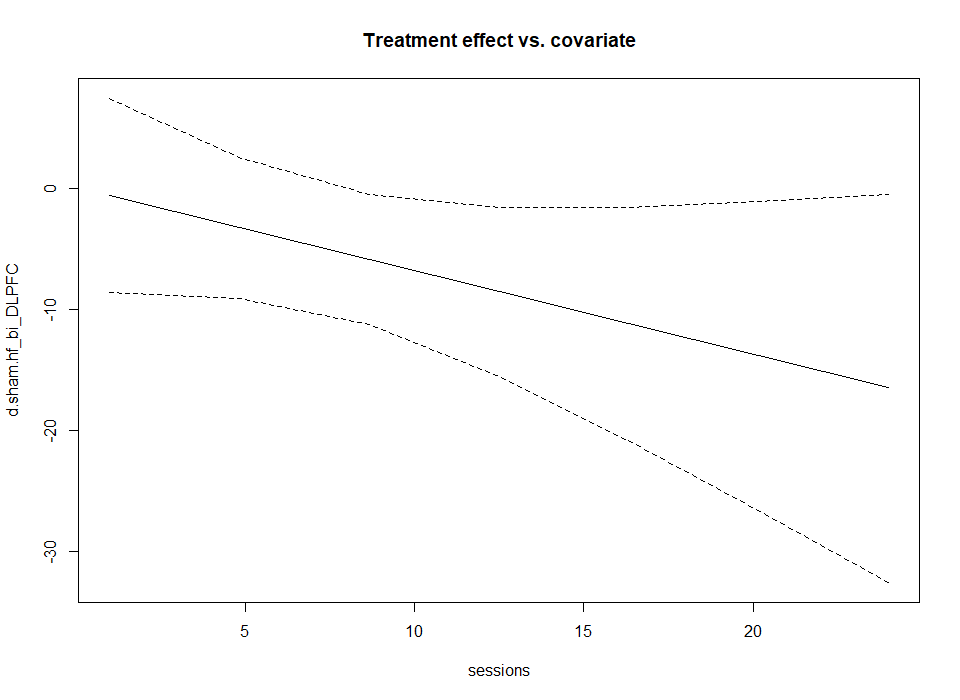
**

**
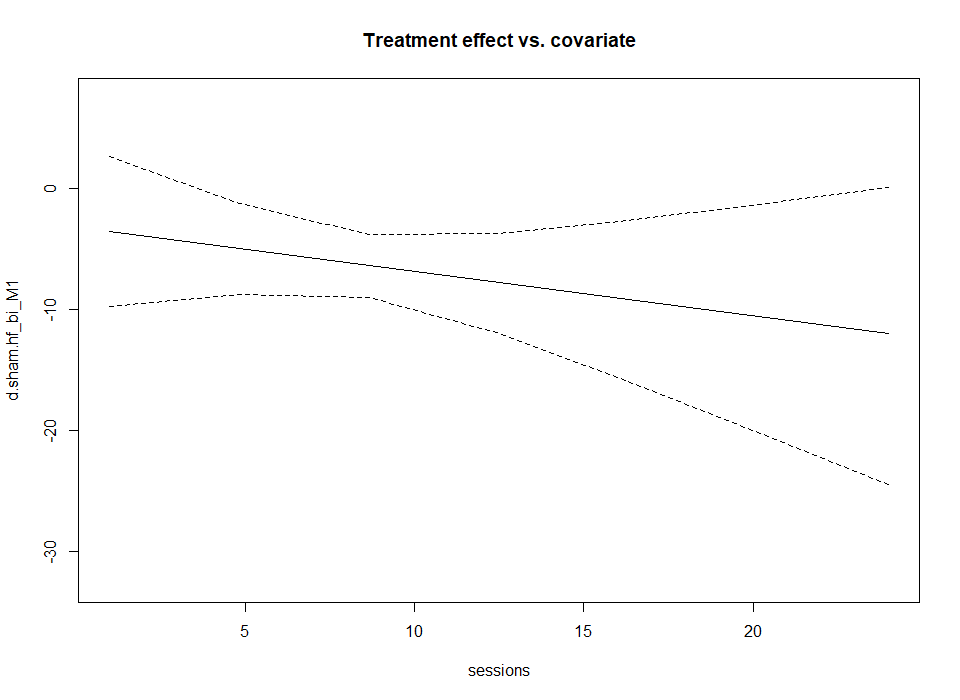
**

# Fig.S6 The funnel plots to assess the bias of publication.


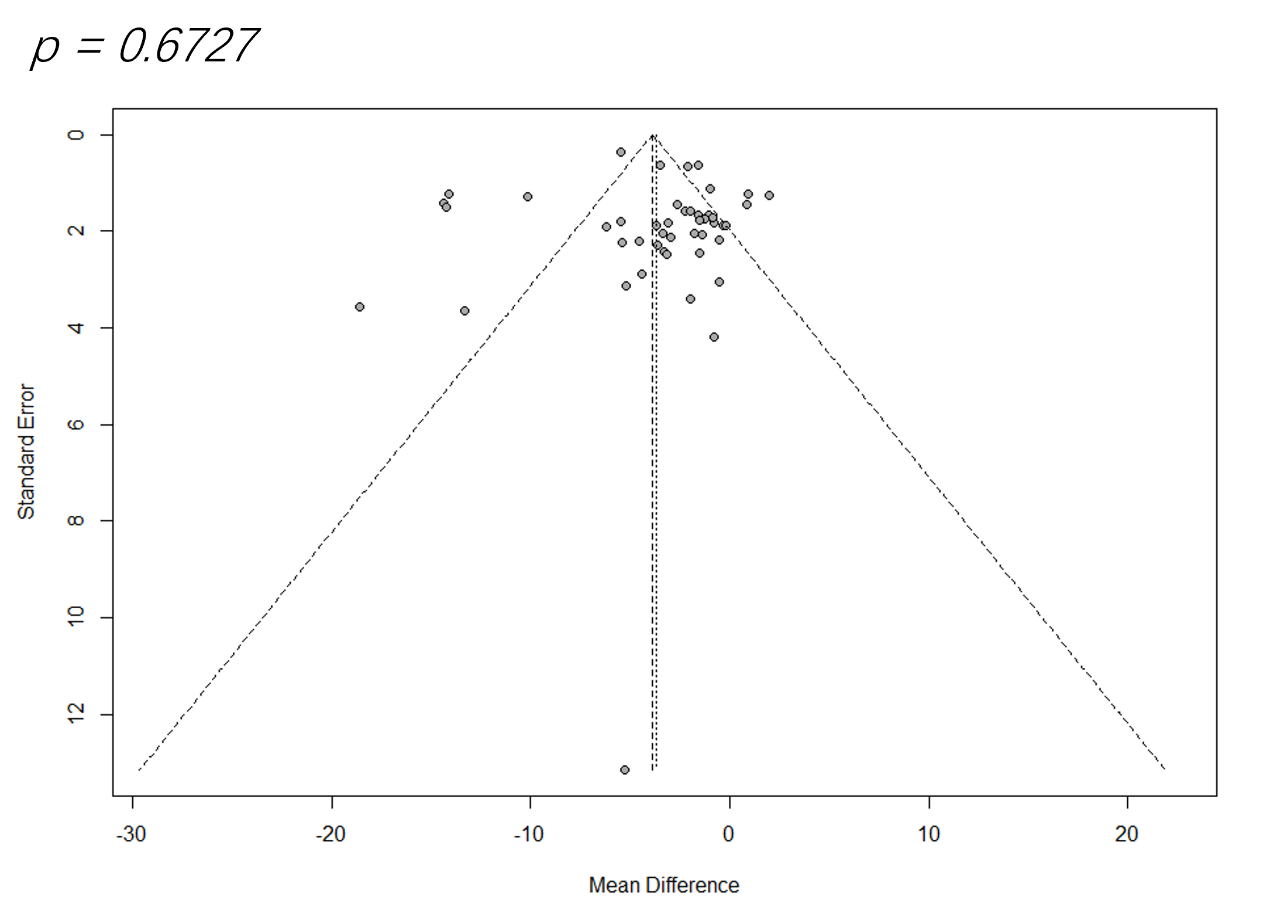

Supplement: Supplementary file 1 [file Data_Sheet_1.docx]
